# Maternal stress in *Shank3^ex4-9^* mice increases pup-directed care and alters brain white matter in male offspring

**DOI:** 10.1371/journal.pone.0224876

**Published:** 2019-11-08

**Authors:** Bibiana K. Y. Wong, Jaclyn B. Murry, Rajesh Ramakrishnan, Fang He, Alfred Balasa, Gary R. Stinnett, Steen E. Pedersen, Robia G. Pautler, Ignatia B. Van den Veyver

**Affiliations:** 1 Department of Obstetrics and Gynecology, Baylor College of Medicine, Houston, Texas, United States of America; 2 Jan and Dan Duncan Neurological Research Institute at Texas Children’s Hospital, Houston, Texas, United States of America; 3 Department of Molecular and Human Genetics, Baylor College of Medicine, Houston, Texas, United States of America; 4 Department of Pediatrics, Section of Pediatrics Neurology and Developmental Neuroscience, Baylor College of Medicine, Houston, Texas, United States of America; 5 Department of Molecular Physiology and Biophysics, Baylor College of Medicine, Houston, Texas, United States of America; Technion Israel Institute of Technology, ISRAEL

## Abstract

Gene-environment interactions contribute to the risk for Autism Spectrum Disorder (ASD). Among environmental factors, prenatal exposure to stress may increase the risk for ASD. To examine if there is an interaction between exposure to maternal stress and reduced dosage or loss of *Shank3*, wild-type (WT), heterozygous (HET) and homozygous (HOM) female mice carrying a deletion of exons four through nine of *Shank3* (*Shank3*^*ex4-9*^) were exposed to chronic unpredictable mild stress (CUMS) from prior to conception throughout gestation. This study examined maternal care of these dams and the white matter microstructure in the brains of their adult male offspring. Overall, our findings suggest that maternal exposure to CUMS increased pup-directed care for dams of all three genotypes. Compared to WT and HET dams, HOM dams also exhibited increased maternal care behaviors with increased time spent in the nest and reduced cage exploration, regardless of exposure to CUMS. Diffusion tensor imaging showed higher mean fractional anisotropy in the hippocampal stratum radiatum of WT and HOM male offspring from dams exposed to CUMS and HOM offspring from unexposed dams, compared to WT male offspring from unexposed dams. These data support that CUMS in *Shank3*-mutant dams results in subtle maternal care alterations and long-lasting changes in the white matter of the hippocampus of their offspring.

## Introduction

Autism Spectrum Disorder (ASD) is a neurodevelopmental disorder characterized by social and communication impairments along with repetitive and stereotypic behaviors [1]. With a prevalence of 1 in 68 children and 1 in 42 in males [2], ASD has become an increasingly important public health concern. The etiology of ASD is multifactorial with highly heterogeneous genetic contribution [3]. Many putative ASD risk genes have been identified [4–6], with major contributing genes those that encode proteins with a role in synaptic function, such as *NRXN1*, *NLGN3*, *NLGN4*, and *SHANK3* [5, 7, 8]. *SHANK3* encodes a predominantly neuronal scaffolding protein that localizes to the postsynaptic density of glutamatergic synapses [9, 10]. The *Shank3*^*ex4-9*^ deletion mice lack all major isoforms of the SHANK3 protein and display abnormal social and repetitive behaviors [11]. Adult *Shank3*^*ex4-9*^ deletion mice have reduced dendritic spine density and deficits in glutamatergic transmission [12]. It has also been described that there are white matter structural abnormalities thought to be associated with social impairment in the brains of individuals with ASD [13].

Although genetic predisposition is a well-established risk factor for the etiology of ASD, recent evidence suggests that environmental factors such as prenatal stress are also associated with an increased risk for ASD [14, 15]. Psychological stress during pregnancy affects a significant number of women, with up to 78% experiencing low-to-moderate levels of antenatal stress [16, 17]. Prenatal stress is also a risk factor for shortened gestation, preterm birth and obstetric complications [18] and has been linked to poor coping with adverse experiences in offspring [19]. Retrospective survey and population-based studies have found a higher incidence of ASD in children born to mothers who experienced more stress during pregnancy [15, 20–25]. In contrast, others found no increased risk for ASD with prenatal adverse life events [26], and even reported that mild to moderate levels of psychological stress can enhance fetal organ and neuromaturation in healthy populations [27, 28]. While the evidence linking ASD risk to increased maternal stress is limited and inconclusive, there is increasing support for the notion that individual variations in neuropsychiatric risk may result from genetic differences that can cause either resilience or vulnerability to environmental stressors [29–31]. Prenatal stress can also affect maternal behaviors, adding another layer of complexity to the impact of the perinatal environment [32].

In this study, we tested in a mouse model the hypothesis that maternal exposure to chronic unpredictable mild stress (CUMS), in the context of a *Shank3* mutation that models an ASD-associated human mutation, influences maternal care behaviors of exposed dams. We also tested if neuronal connectivity in relevant brain regions in exposed offspring was altered.

## Materials and methods

### Mice

All methods and animal care procedures were approved by Baylor College of Medicine (BCM) Institutional Animal Care and Use Committee (IACUC). The approved protocol number is AN-5888. All experiments were conducted according to institutional and governmental regulations concerning the ethical use of animals in research. The animal facilities are approved by the Association for Assessment and Accreditation for Laboratory Animal Care International (AAALAC). Animals were housed in AAALAC approved Transgenic Mouse Facility (TMF) at BCM. All maternal behavioral testing were conducted in the Neurobehavioral core facility of the BCM Intellectual and Developmental Disabilities Research Center (IDDRC) situated in the TMF. All experimental animals were generated by mating heterozygous (HET) *Shank3*^*ex4-9*^ *(Shank3*^*tm1Yhj*^, *JAX # 017442)* male and female mice, generously donated by Dr. Arthur Beaudet (Baylor College of Medicine, Houston, TX). The mouse line was maintained on a C57BL/6J background. Standard rodent chow (Pico LabRodent Diet, #5053, Purina USA) and water were available *ad libitum*. The colony room was maintained on a 12∶12 light/dark cycle with lights on at 07∶00 hour. Genotyping of *Shank3*^*ex4-9*^ mice was performed per Wang *et al*. 2011 [33] with minor modifications. A group of mice comprising of 6–13 dams per genotype and CUMS exposure were examined for maternal behavior. All behavioral testing were performed during the light period (09:00–17:00 hour) and mice were acclimated to the testing rooms for ≥ 30 minutes prior to each assay.

Detailed materials and methods including mouse breeding strategy, CUMS protocol, genotyping, and exact number of mice used for each test are provided in S1 File (Supplemental materials and methods).

### Maternal care behavioral testing

Maternal care behavioral testing was conducted during the first eight days postpartum (PND 1–8) on wildtype (WT), heterozygous (HET), and homozygous (HOM) *Shank3*^*ex4-9*^ adult females who were at least six weeks old. These dams had either been exposed to chronic unpredictable mild stress (CUMS) from one week before mating with HET sires until just prior to parturition or were unexposed to CUMS (Control). The order of testing was as follows and was the same for all dams tested: nesting quality scores (PND 1 and 3), maternal in-nest care assessments (PND 1, 3, and 5), pup retrieval test (PND 3), and maternal intruder test (PND 8) [34–37]. For the maternal in-nest care assessments, pup retrieval, and maternal intruder tests, videos were recorded under room lighting in the home cage with a Plexiglas lid in a biological fume hood with the fan running to produce background noise. During data acquisition, the test administrator was blinded to genotype and exposure, and videos were de-identified. Videos were scored using a Psion 5.0 hand-held computer, and data was extracted using the Noldus Observer (Leesburg, VA). Details of individual tests are provided in S1 File (Supplemental materials and methods).

### Diffusion tensor imaging

Diffusion tensor imaging (DTI) was performed on eight-week-old WT and HOM adult male offspring from control and CUMS-exposed *Shank3*^*ex4-9*^ HET dams (Control: 5 WT, 4 HOM; CUMS: 2 WT, 3 HOM). The mice were anesthesized with isoflourane (Cat# NDC 11695-6776-2, Henry Schein Animal Health, Dublin, OH) and transcardially perfused with heparinized phosphate buffered saline (PBS) followed by fixation with 4% paraformaldehyde (PFA). Perfusion was carried out using a perfusion pressurization set up from Warner Instruments (Model VPP-6, Hamden, CT). The skulls were exposed, fixed in PFA and stored in 5mM gadopentate dimeglumine (Cat# NDC 50419-188-15; Bayer, Leverkusen, Germany) at 4°C until imaging was performed to assess for white matter tractography in the brains [38]. For structural characterization of offspring brains, whole brain voxel-voxel analysis was applied, and the average fractional anisotropy (FA) values in regions of interest (ROI) were reported. Further details of image acquisition and data processing are in S1 File (Supplemental materials and methods).

### Statistical analysis

All data were analyzed by a two-way ANOVA for main effects of genotype (G) and exposure (E) and for gene × exposure interaction (G×E) using Prism GraphPad Version 6 (La Jolla, CA). Significant main effects and interactions were followed up with Tukey’s post hoc analysis for multiple comparisons. Data are presented as mean ± standard error of the means (SEM) and the statistical significance was set at *P* < 0.05.

## Results

### CUMS exposure does not significantly affect pregnancy outcomes of *Shank3*^*ex4-9*^ mutant dams

**Fig 1:**
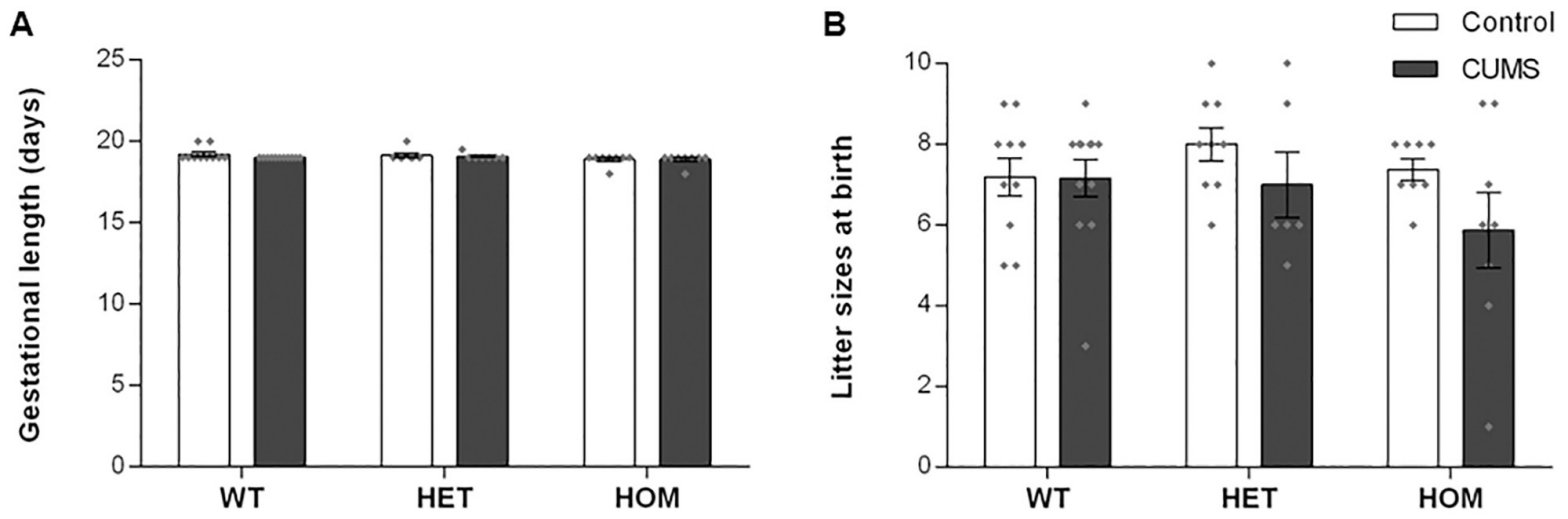
CUMS exposure in *Shank3*^*ex4-9*^ mutant dams did not affect pregnancy outcomes. (A) Gestational length, and, (B) litter size at birth were not affected by *Shank3*^*ex4-9*^ genotypes and CUMS exposure. WT: Wildtype; HET: Heterozygous; HOM: Homozygous. N for gestational length = Control (10 WT, 7 HET and 8 HOM) and CUMS (10 WT, 6 HET and 8 HOM). N for litter sizes = Control (10 WT, 9 HET, and 8 HOM) and CUMS (12 WT, 6 HET and 8 HOM). Individual data points are represented by diamonds. Bars are mean ± SEM and P<0.05 statistically significant by 2-way ANOVA.

To evaluate the impact of preconceptional and gestational exposure to CUMS on pregnancy outcomes, we assessed gestational length and litter size of WT, HET and HOM *Shank3*^*ex4-9*^ mutant dams. Gestational length (Fig 1A) was not affected by genotype (F_2,45_ = 2.902, ns) and CUMS exposure (F_1,45_ = 1.032, ns), and there was no G×E interaction (F_2,45_ = 0.484, ns). Similarly, there was no main effect of genotype (F_2,47_ = 1.080, ns) and exposure (F_1,47_ = 3.213, ns), and no G×E interaction (F_2,47_ = 0.939, ns) on litter size at birth (Fig 1B). These findings suggest that the maternal *Shank3* genotype and maternal exposure to CUMS did not affect pregnancy outcomes.

### Exposure to CUMS increases pup-directed care and *Shank3*^*ex4-9*^ mutant dams exhibit more maternal in-nest care behaviors

**Fig 2:**
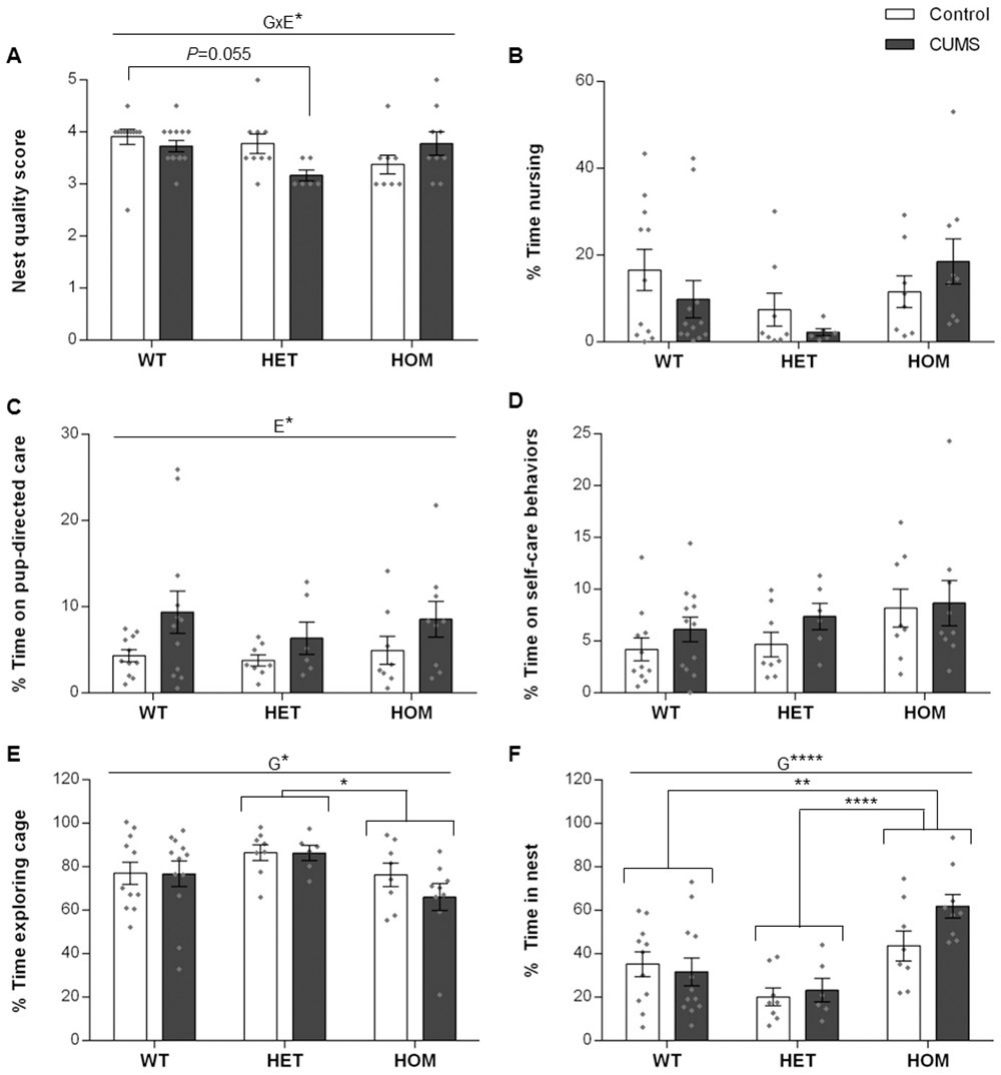
Exposure to CUMS increased pup-directed care and *Shank3*^*ex4-9*^ mutant dams exhibited more maternal in-nest care behaviors. (A): Nest quality scores. A significant G×E interaction was observed, likely driven by a lower nest quality in CUMS-exposed HET dams; (B): Percent time dams spent nursing. Genotype and exposure did not affect the amount of nursing time spent by dams; (C) Percent time dams engaged in pup-directed care behaviors. CUMS exposure significantly increased the amount of time dams spent on pup-directed behaviors; (D): Percent time that dams spent engaging in self-care. CUMS exposure or genotype did not affect the time dams spent in self-care; (E): Time spent in cage exploration. CUMS exposure did not alter the time dams spent in cage exploration, but there was a significant genotype effect with HOM dams spending less time exploring their cage compared to HET dams; (F): Percent time in nest. HOM dams spent more time in the nest compared to WT and HET dams, and CUMS exposure did not affect this difference. WT: Wildtype; HET: Heterozygous; HOM: Homozygous; N for nest quality = Control (11 WT, 9 HET, and 8 HOM) and CUMS (13 WT, 6 HET and 9 HOM). N for maternal care: Control (11 WT, 8 HET, and 8 HOM) and CUMS (12 WT, 6 HET, and 9 HOM). G: Main effect of genotype, E: Main effect of exposure, G×E: Genotype × exposure interaction; Individual data points are represented by diamonds. *P < 0.05, **P < 0.005; ****P < 0.0001.

Maternal care behaviors, including nest building, nursing and grooming are necessary for pup survival [11]. It has been reported that mild chronic stress increases nursing and grooming but does not modulate nesting or nurturing behaviors, while ultra-mild chronic stress does not alter maternal care [39, 40]. There was a significant G×E interaction on nest quality scores (Fig 2A; F_2,50_ = 4.079, *P* < 0.05). Post-hoc analysis identified a statistical trend (*P* = 0.055) of lower quality nest built by HET *Shank3*^*ex4-9*^ dams exposed to CUMS compared to nests built by unstressed WT dams. There were no differences in nest qualities scores between all other groups.

The time spent nursing was unaffected by genotype (Fig 2B, G: F_2,48_ = 2.522, *P* = 0.09). CUMS exposure increased pup-directed care behaviors of dams across all genotypes (Fig 2C; E: F_1,48_ = 6.126, *P* < 0.05), while the *Shank3* genotype did not change pup-directed care behaviors (Fig 2C; G: F_2,48_ = 0.530, ns).

Self-care behaviors of dams was not affected by CUMS exposure (Fig 2D; E: F_1,48_ = 1.807, ns; G×E: F_2,48_ = 0.236, ns), or by genotype (F_2,48_ = 2.571, *P* = 0.087). The amount of time that dams spent exploring their cages was also not altered by CUMS exposure (Fig 2E; E: F_1,48_ = 0.6016, ns; G×E: F_2,48_ = 0.536, ns), but there was a genotype effect (F_2,48_ = 3.332, *P* < 0.05) with HOM dams spending less time exploring their cages compared to HET *Shank3*^*ex4-9*^ dams (Post-hoc *P* < 0.05).

*Shank3*^*ex4-9*^ HOM dams also spent more time in the nest compared to WT and *Shank3*^*ex4-9*^ HET dams (Fig 2F, G: F_2,48_ = 12.03, *P* < 0.0001; post-hoc *P* < 0.003), and this was not affected by exposure to CUMS (E: F_1,48_ = 1.428, ns; G×E: F_2,48_ = 1.842, ns).

### CUMS exposure of *Shank3*^*ex4-9*^ mutant dams does not alter their pup retrieval and maternal aggression responses to intruders

**Fig 3:**
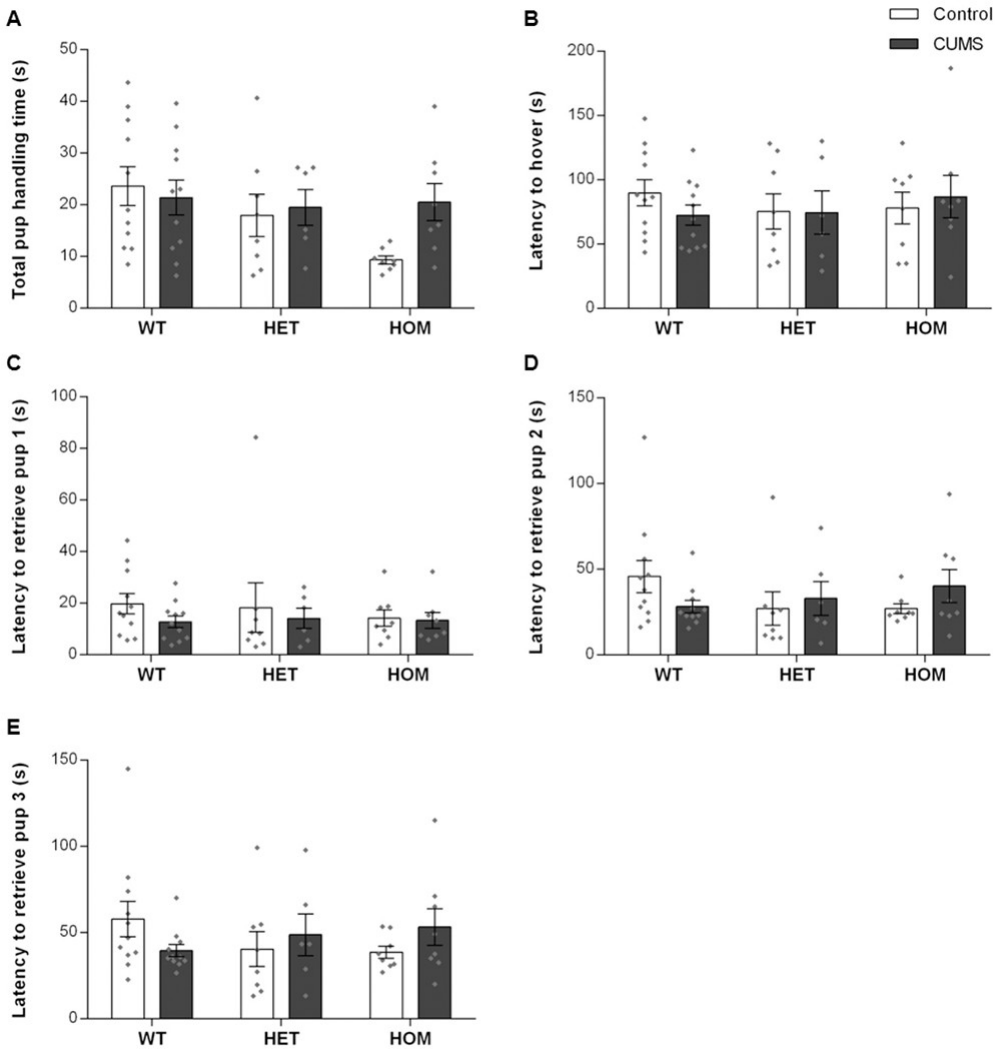
CUMS exposed did not alter pup retrieval responses in *Shank3*^*ex4-9*^ mutant dams. (A): Total time dams spent handling pups. (B): Latency for dams to hover over pups in the nest. (C, D, E): Latency for dams to retrieve their 1^st^, 2^nd^, and 3^rd^ pups, respectively, and return them to the nest. CUMS exposure and genotypes did not affect any of the pup retrieval parameters. WT: Wildtype; HET: Heterozygous; HOM: Homozygous. N = Control (11 WT, 8 HET, and 8 HOM) and CUMS (12 WT, 6 HET, and 8 HOM). Individual data points are represented by diamonds.

Studies have shown that maternal exposure to mild stress can robustly increase the pup retrieval response, but that exposure to ultra-mild stress increases the latency to first pup retrieval [39, 40]. In our study, no observable differences were identified in total pup handling time (Fig 3A; G: F_2,46_ = 2.541, ns; E: F_1,46_ = 1.411, ns; G×E: F_2,46_ = 1.842, ns), or in the time it took dams to crouch or hover over the nest while all pups were in the nest (Fig 3B; G: F_2,46_ = 0.1779, ns; E: F_1,46_ = 0.0951, ns; G×E: F_2,46_ = 0.6203, ns). When pups were removed from the nest and placed in a corner of the cage, all dams took a similar amount of time to collect the pups and place them back in the nest, irrespective of genotype or exposure to CUMS (Fig 3C–3E; ns).

**Fig 4:**
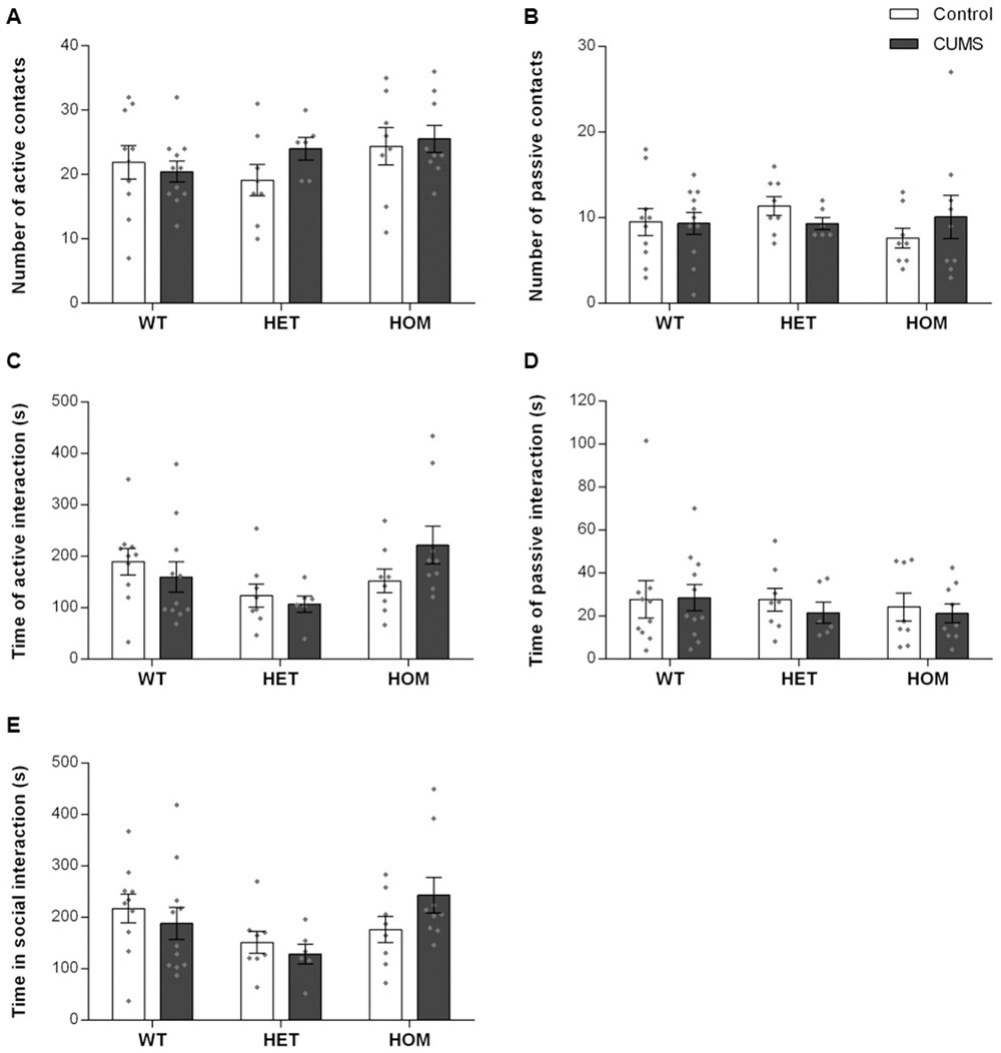
CUMS exposure did not alter maternal aggression responses to intruders in *Shank3*^*ex4-9*^ mutant dams. (A, B): Number of active (A) and passive (B) social contacts by dams during maternal intruder test. (C, D): Time dams spent in active (C) and passive (D) social interaction with the intruder. (E): Time spent in social activities during maternal intruder test. CUMS exposure and genotypes did not affect any of the parameters assessed during the maternal intruder test. WT: Wildtype; HET: Heterozygous; HOM: Homozygous. N = Control (11 WT, 8 HET, and 8 HOM) and CUMS (11 WT, 6 HET, and 9 HOM). Individual data points are represented by diamonds.

Gestational stress can also result in altered maternal responses towards an intruder manifesting as either heightened aggression or impaired defense behaviors depending on the severity of the stress paradigm [39, 40]. There were no differences between groups in the number of and time spent on active or passive contacts (Fig 4A–4D; ns), or in total time engaged in social interactions (Fig 4E; G: F_2,46_ = 2.988, ns; E: F_1,46_ = 0.04353, ns; G×E: F_2,46_ = 1.672, ns).

### Fractional anisotropy of white matter is significantly altered in brains of offspring from heterozygous *Shank3*^*ex4-9*^ mutant dams exposed to CUMS

**Fig 5:**
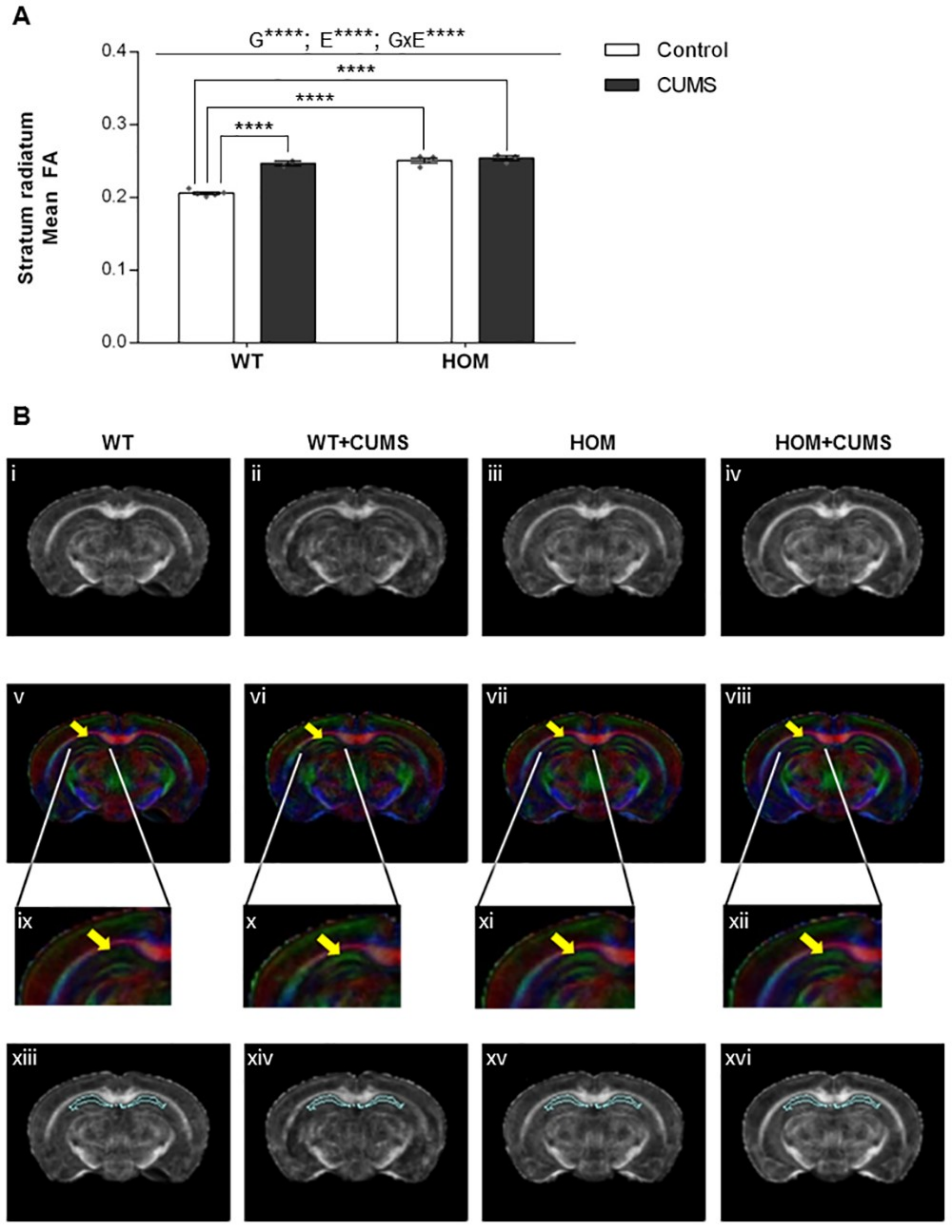
DTI analysis showed significant white matter alteration in the stratum radiatum of the hippocampus in WT male offspring of CUMS-exposed dams and in HOM offspring. (A): Fractional anisotropy (FA) values for the stratum radiatum. WT offspring of CUMS-exposed dams and all HOM offspring showed increased FA values compared to WT offspring of control dams. (B): Representative coronal views depicting the FA values (i-iv) and direction-encoded color maps (DECs) that were generated across the representative coronal views (v-viii). The yellow arrow on the DEC images highlights the stratum radiatum, the identified region with differential mean FA values between the groups, which is enlarged in panels ix-xii. Panels xiii-xvi show outlines of the stratum radiatum overlaid on the FA images. Each column represents a group, from left to right: Control WT (Row 1; panels i, v, ix, xii), CUMS WT (Row 2; panels ii, vi, x, xiv), Control HOM (Row 3, panels iii, vii, xi, xv) and CUMS HOM (Row 4; panels iv, viii, xii, xvi). WT: Wildtype; HOM: Homozygous. N = Control (5 WT, 4 HOM) and CUMS (2 WT, 3 HOM). G: Main effect of genotype, E: Main effect of exposure, G×E: Genotype × exposure interaction; Individual data points in panel A are represented by diamonds.****P < 0.0001.

Maternal stress can cause changes in white matter in brains of offspring that can persist into adulthood [41]. Additionally, there is DTI evidence of white matter structural abnormalities in children with ASD that is associated with social impairment [13, 42]. Adult WT and HOM male offspring were examined by DTI and the fractional anisotropy (FA) values compared. A voxel by voxel analysis was used to evaluate 29 brain regions of interest based on the Hopkins mouse brain template (S1 Table). Compared to the unexposed control group, WT male offspring from dams exposed to CUMS had increased mean FA values in the stratum radiatum, a distinct region of the CA1 area in the hippocampus (Fig 5A; G: F_1,10_ = 72.61, *P* < 0.0001; E: F_1,10_ = 52.10, *P* < 0.0001; G×E: F_1,10_ = 38.97, *P* < 0.0001; post-hoc P < 0.0001). This CUMS-associated increase in stratum radiatum FA value was not observed in HOM male offspring (Fig 5A; post-hoc P > 0.5). The FA changes in the stratum radiatum (Fig 5B, i-iv) can be seen in the direction-encoded color maps (DEC) (Fig 5B, v-viii), the zoomed-in images for the DEC panel (Fig 5B, ix-xii) and the outline of the stratum radiatum segmentation (Fig 5B, xiii-xvi) from representative coronal images comparing adult WT and HOM male offspring with and without CUMS exposure. These data show potential changes in directionality and diffusion in this contiguous region (Fig 5B, ix-xii; yellow arrow).

Amongst the other examined brain regions, the neocortex and nucleus accumbens of offspring exposed to maternal CUMS had reduced mean FA values irrespective of the *Shank3* genotype indicating an effect of stress on these regions (S1 Fig: Neocortex: G: F_1,10_ = 0.707, ns; E: F_1,10_ = 9.446, *P* < 0.05; G×E: F_1,10_ = 1.103, ns; Nucleus accumbens: G: F_1,10_ = 2.663, ns; E: F_1,10_ = 7.517, *P* < 0.05; G×E: F_1,10_ = 0.00851, ns).

HOM males showed higher mean FA values in the amygdala and septum irrespective of exposure to maternal CUMS (S1 Fig: Amygdala: G: F_1,10_ = 4.974, *P* < 0.05; E: F_1,10_ = 3.015, ns; G×E: F_1,10_ = 1.790, ns; Septum: G: F_1,10_ = 6.891, *P* < 0.05; E: F_1,10_ = 0.5451, ns; G×E: F_1,10_ = 1.064, ns). In the piriform cortex, stress exposure and genotype both showed a reduction in mean FA values (S1 Fig: G: F_1,10_ = 6.08, *P* < 0.05; E: F_1,10_ = 9.648, *P* < 0.05; G×E: F_1,10_ = 0.408, ns).

## Discussion

Through the study of numerous mouse models carrying different *Shank3* alleles, from complete disruption of the locus to specific inactivation of the major full-length isoforms, it is clear that disruption of *Shank3* in mice causes varying degrees of ASD-like behaviors and synaptic dysfunction [33, 43–46]. However, the incomplete penetrance of *SHANK3* mutations in humans suggests that there may be non-genetic contributing factors as well as modifier loci. Given the conflicting reports on the effects of maternal stress as a risk factor for ASD in humans [15, 20–26] and the limited ability of human studies to control or account for parental genotypes, presence of underlying maternal psychiatric illness, and presence of adverse postnatal environments [15], we sought to evaluate the effect of CUMS exposure during pregnancy in an isogenic mouse model.

The *Shank3*^*ex4-9*^ mutant mouse is a well-characterized model of human ASD, with HOM mice exhibiting abnormal social and repetitive behaviors [33, 43–45, 47]. This study is the first to evaluate the direct effects of CUMS exposure on maternal behaviors of dams in relationship to their *Shank3*^*ex4-9*^ genotype.

With respect to stress, we found that CUMS had mild effect on maternal behaviors of dams of all *Shank3*^*ex4-9*^ genotypes consisting only of increased time spent on pup-directed care. There was also a decrease in nest quality score for CUMS-exposed HET dams, but all other measures of maternal care (i.e. time spent nursing, pup retrieval, and maternal aggressive behaviors) and pregnancy outcome (i.e. gestational length and litter size) were not altered by CUMS exposure. Most of our findings are consistent with previous reports that mild to ultra-mild prenatal stress did not alter nest building or nurturing behaviors [39, 40], but we could not replicate the observation by Meek *et al*. [39] that exposure to mild stress in Swiss-Webster mice, which are not known to be more sensitive to stress [48, 49], robustly increased the pup retrieval response. In contrast, our observation of increased pup-directed behavior in the CUMS group was consistent with findings from Maestripieri *et al*. [50], who described a positive correlation between prenatal chronic stress-induced anxiety and increased maternal care behaviors. The overall findings likely differed between these studies because they used a slightly different stress protocol, indicating that even minor changes in the timing and types of applied environmental stressors modifies how maternal care behavior is affected.

Since the *Shank3*^*ex4-9*^ mutant mouse is a model for ASD, previous studies of this model have not focused on maternal care behavior. Here we demonstrate that *Shank3*^*ex4-9*^ mutation does not negatively impact maternal care of dams. In addition to more CUMS-induced pup-directed behavior, *Shank3*^*ex4-9*^ HOM dams spent more time in the nest and less time in cage exploration also suggesting more nurturing behavior. The lower cage exploration is not because *Shank3*^*ex4-9*^ HOM females are less active, as Wang *et al*. showed that they have typical locomotion in the open field test [33]. *Shank3*^*ex4-9*^ HOM mice also investigate novel objects more often from within than from outside the nest, which is not due to increased anxiety levels as these mice perform the same as WT mice on anxiety tests [33]. This finding combined with other behavioral anomalies they observed led Wang *et al*. to suggest that *Shank3*^*ex4-9*^ HOM mice have less behavioral plasticity, which may model impairments in novelty processing associated with human ASD [33, 51]. Therefore, although their increased time spent in the nest and lower cage exploration seem to suggest that *Shank3*^*ex4-9*^ HOM dams are better caregivers to their pups, these behavioral changes may actually not be driven by desire to care for their pups.

We and others have postulated that gene-environment interactions modulate the severity of the ASD phenotype [7]. Data in rodent models indicate that chronic prenatal stress alters brain development with the severity of changes being influenced by genetic background [29]. Prenatal stress alters development of the hippocampus, prefrontal cortex and amygdala, causing decreased neurogenesis and neuronal connectivity [29]. In addition to synaptic dysfunction, abnormal development of neural connectivity is also observed in children with ASD and has been implicated in pathologies of social behavior [41, 52–54]. The majority of DTI studies on ASD brains in humans and in animal models have found FA value changes in various brain regions that rarely overlap between individuals, but changes in either direction have been correlated with the severity of ASD symptoms [52]. We found that prenatal CUMS exposure significantly increased FA of the stratum radiatum, located in the CA1 region of the hippocampus, but only in WT males with FA values similar to those observed in HOM males with and without prenatal CUMS exposure. In previous characterizations of different *Shank3* knockout mouse models, there were fewer hippocampal CA1 pyramidal neurons with altered neurite structure and lower current amplitude. In addition, impaired neuroplasticity, as measured by long-term potentiation (LTP), is also predominantly observed in the stratum radiatum [43, 55]. Similarly, mice with a mutation in *NLGN3*, another synaptic protein linked to ASD, also have altered synaptic structures in the stratum radiatum [56], thus these mouse models share structural changes in the stratum radiatum and social interaction deficits [43, 56].

In addition to the CUMS-associated FA changes in the WT stratum radiatum, we also found lower FA values in the neocortex and nucleus accumbens in the CUMS group, irrespective of the *Shank3*^*ex4-9*^ genotype. The changes in FA in the neocortex are consistent with previous reports that prenatal stress alters the medial prefrontal cortex, a region within the neocortex [57]. In a large human case-control study, ASD was associated with lower volume of nucleus accumbens [58]. The nucleus accumbens also plays a role in the mesolimbic reward pathway with connections to the amygdala [59], where we observed a significant genotype effect with an increased FA value in HOM males compared with WT mice, regardless of CUMS exposure. Although it is currently unclear through which mechanism these structural alterations correlate with social behavior in the context of prenatal stress and the *Shank3* genotype, the affected regions have previously been identified as abnormal in individuals with ASD or have been associated with social deficits in humans and mouse models. Finally, we detected significant effects of CUMS exposure and *Shank3*^*ex4-9*^ genotype, but no G×E interaction on FA values, in the piriform cortex, a region susceptible to structural changes upon stress [60].

In summary, our data indicate that perinatal maternal exposure to chronic mild stress influences maternal behavior. We found that CUMS-exposed dams have more pup-directed care behaviors, the HOM *Shank3* mutation caused dams to spend more time in the nest and less time exploring the cage, suggestive of increased maternal care behaviors. Furthermore, in male offspring we correlated FA changes in the stratum radiatum of the hippocampal CA1 region, with exposure to maternal CUMS in WT mice leading to white matter connectivity that mimics that of *Shank3*^*ex4-9*^ HOM mice. Characterization of this *Shank3* mutant mouse, a genetic mouse model of ASD, in the context of a chronic mild stress paradigm allowed us to determine that incremental burden of an adverse environmental exposure contributes to the severity of some maternal behaviors and alterations in brain white matter tractography.

## Supporting information

S1 Data Supplemental datatables. These are the accompanying supplemental datatables that for the data used to generate all figures. Excel sheet labels correspond to each figure panel.
(XLSX)

S1 Fig DTI analysis in the white matter of male offspring from CUMS-exposed dams. Fractional anisotropy (FA) values for the indicated brain regions in WT and HOM male offspring of CUMS-exposed dams are presented. CUMS exposure reduced FA values in both the neocortex (A) and nucleus accumbens (B), regardless of genotypes. There was an increase in FA values in the amygdala (C) and septum (D) of male offspring of all three genotypes, irrespective of CUMS exposure. There were also significant main effects of genotype and exposure in the piriform cortex, with higher FA values in HOM compared to WT; CUMS exposure reduced the FA values in both groups. WT: Wildtype; HOM: Homozygous. N = Control (5 WT, 4 HOM) and CUMS (2 WT, 3 HOM). Individual data points are represented by diamonds. G: Main effect of genotype, E: Main effect of exposure; *P < 0.05.
(TIF)

S1 File Supplemental materials and methods. This file contains all supplemental materials and methods.
(DOCX)

S1 Table Most offspring brain regions analyzed by DTI do not show significant differences between groups. Results of diffusion tensor imaging (DTI) conducted in 24 additional brain regions of offspring from HET dams with and without CUMS exposure (Control/CUMS). The number of male offspring in each group was Control (5 WT, 4 HOM) and CUMS (2 WT, 3 HOM).
(DOCX)
