# Supplementary figures and images for: Maternal stress in Shank3ex4-9 mice increases pup-directed care and alters brain white matter in male offspring
Source: PLoS One. 2019 Nov 8;14(11):e0224876. doi: 10.1371/journal.pone.0224876 (PMC6839842; doi:10.1371/journal.pone.0224876)

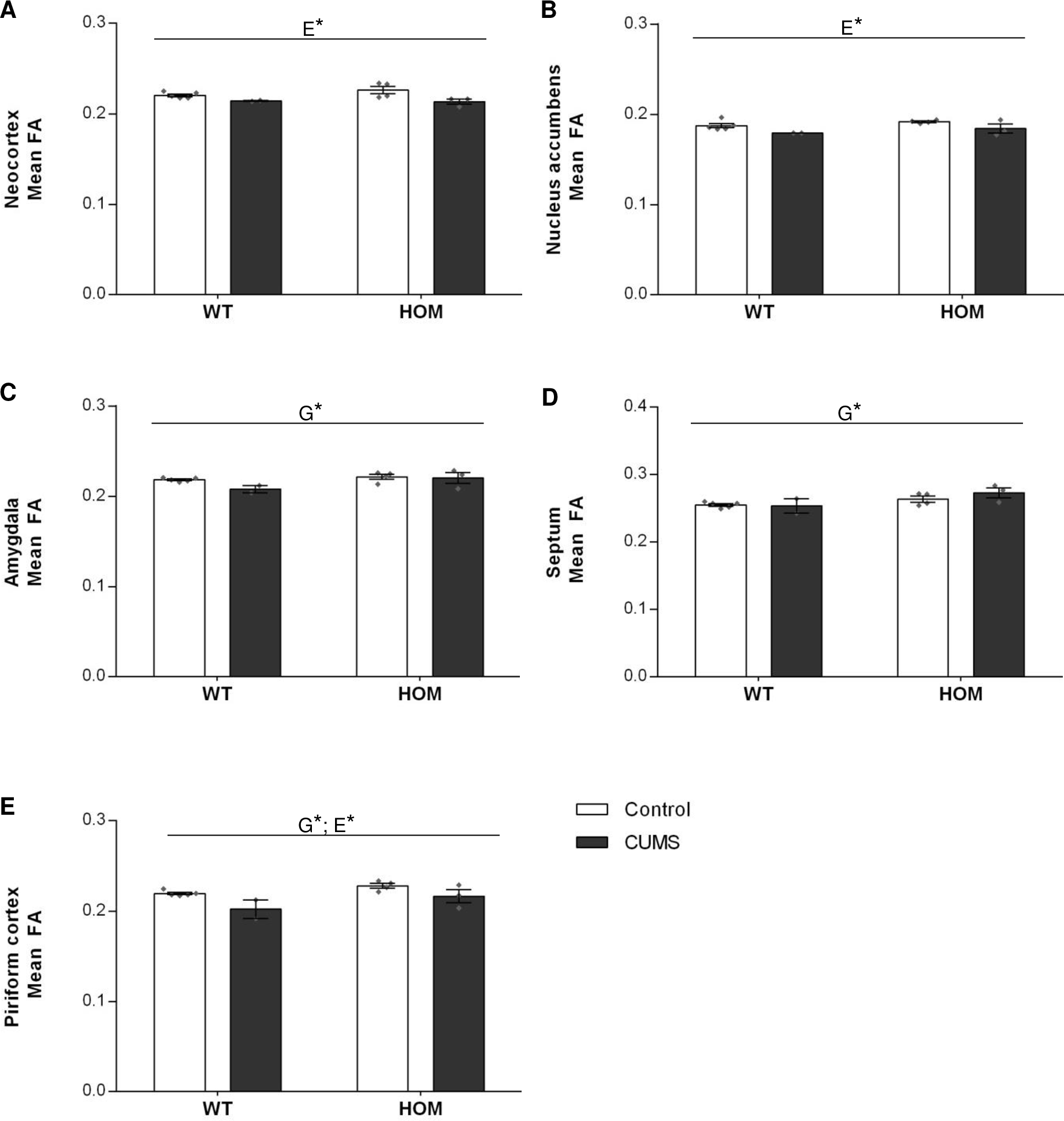

Supplement: S1 Fig — Fractional anisotropy (FA) values for the indicated brain regions in WT and HOM male offspring of CUMS-exposed dams are presented. CUMS exposure reduced FA values in both the neocortex (A) and nucleus accumbens (B), regardless of genotypes. There was an increase in FA values in the amygdala (C) and septum (D) of male offspring of all three genotypes, irrespective of CUMS exposure. There were also significant main effects of genotype and exposure in the piriform cortex, with higher FA values in HOM compared to WT; CUMS exposure reduced the FA values in both groups. WT: Wildtype; HOM: Homozygous. N = Control (5 WT, 4 HOM) and CUMS (2 WT, 3 HOM). Individual data points are represented by diamonds. G: Main effect of genotype, E: Main effect of exposure; *P < 0.05. (TIF) [file pone.0224876.s002.tif]
