# Supplementary material for: Maternal stress in Shank3ex4-9 mice increases pup-directed care and alters brain white matter in male offspring
Source: PLoS One. 2019 Nov 8;14(11):e0224876. doi: 10.1371/journal.pone.0224876 (PMC6839842; doi:10.1371/journal.pone.0224876)

**Supplemental Materials and Methods**

**Mice Breeding & CUMS protocol**

Nulliparous females of all three genotypes, wild-type (WT), heterozygous (HET), and homozygous (HOM), were socially housed and were randomly assigned to exposure groups: chronic unpredictable mild stress (CUMS) or no added stress (control) one week before conception. Dams in the CUMS group were subjected to a biweekly combination of diurnal and nocturnal chronic unpredictable mild stressors that were modified for preconception and prenatal CUMS exposure use [[1](#_ENREF_1), [2](#_ENREF_2)]. The diurnal stressors were cage soiling (50 mL of water added to the cage), cage tilt (30°), and confinement (plastic ware container with holes). Nocturnal stressors included overnight light, cage tilt and cage soiling. The diurnal stressors were administered for two hours, after which a one to two-hour rest period was given. Then the nocturnal stressor was initiated, maintained throughout the night, and stopped the following morning. This pattern was continued until late gestation but prior to parturition (E17.5 ± 2 days).

**Genotyping**

DNA for PCR analysis was prepared by immersing tails in 500 uL of lysis buffer (50 mM NaOH) for 30 minutes at 100°C. Tubes were vortexed and allowed to cool for 10 minutes at room temperature. 50 uL of 1 M TrisCl was added to neutralize the NaOH, and the contents mixed thoroughly and centrifuged at 15,000 rpm for 5 minutes. For every PCR reactions, 2 uL of the supernatant was used with 100 uM primers for each genotype. To determine genotypes, two PCR reactions were run in parallel: Reaction 1 uses 35 cycles with 62°C annealing temperature to amplify a 700 bp fragment corresponding to the wild-type allele using primers:

*Shank3*-F: 5´-GTATAGTGTCTTTGCATCTGGGAC-3´ and

*Shank3*-R: 5´-AAAGTTTCACTGACTGGCCTTGTC-3´.

Reaction 2 uses 30 cycles with 60°C annealing temperature to amplify a 400 bp fragment corresponding to the mutant allele using primers:

*Shank3*-MF: 5´-GTATAGTGTCTTTGCATCTGGGAC-3´ and

*Shank3*-MR: 5´- GTGGGCTCTATGGCTTCTGA-3´.

**Perinatal outcomes**

To assess the early life outcomes for litters of WT, HET and HOM dams with and without exposure (CUMS or Control), we compared the mean duration of pregnancy and the mean litter sizes at birth (PND 0). We evaluated gestational length for 10 WT, 7 HET and 8 HOM control dams and 10 WT, 6 HET and 8 HOM CUMS dams. Mean litter sizes were assessed for 10 WT, 9 HET, and 8 HOM control dams and 12 WT, 6 HET and 8 HOM CUMS dams.

**Nest quality scores**

A clean, cotton nestlet square was put in the home cage as nesting material for each dam. To evaluate the quality of the nest built by dams with and without exposure during the early postpartum period, we assigned the nest quality a score of 1 through 5 ranging from an untouched nestlet, a partially torn up nestlet, mostly shredded nestlet, an identifiable but flat nest, and a (near) perfect nest [[4](#_ENREF_4)] that was averaged across two early postpartum days (PND 1, and 3) [[5](#_ENREF_5)]. We evaluated nest quality scores for 11 WT, 9 HET, and 8 HOM control dams and 13 WT, 6 HET and 9 HOM CUMS dams.

**Maternal care assessments**

Maternal behavior was recorded across three 10-minute observation periods in the home cage on PND 1, 3, and 5 [[5](#_ENREF_5), [6](#_ENREF_6)]. Maternal care behaviors were scored and analyzed as percent time spent in pup-directed behaviors (nursing) and pup-attentive behaviors (licking or handling) versus non-maternal behaviors: self-care such as, eating, drinking, grooming, or cage exploratory behaviors. Proximity to the nest locations (in nest time) was also recorded. These behaviors were scored with the video playback set at half speed to allow for simultaneous, continuous assessments for multiple types of maternal care measurements across the total 30-minute test. We evaluated maternal care for 11 WT, 8 HET, and 8 HOM control dams and 12 WT, 6 HET, and 9 HOM CUMS dams.

**Pup retrieval**

A 5-minute pup retrieval test was conducted on PND 3 in the home cage [[7](#_ENREF_7)]. Three pups were selected at random, removed from the nest, and placed on the opposite side of the home cage. Pup retrieval videos were scored for latency in seconds (s) for dams to pick up pups and return them to the nest (1st, 2nd, and 3rd pups). Latency to first crouching over the pups or hovering in the nest, in seconds (s), and the total time that dams spent handling pups was also scored. We evaluated pup retrieval in 11 WT, 8 HET, and 8 HOM control dams and 12 WT, 6 HET, and 8 HOM CUMS dams.

**Maternal intruder test**

A 10-minute test was conducted on PND 8 in the home cage [[8](#_ENREF_8)]. Novel interaction partners, which were age-matched and conspecific virgin females with marked tails were introduced into the home cage on the opposite side of the nest. Responses of subject dams were scored for the number of events and the total time (s) engaged in active social interactions (defined as when the subject dam initiated contact with the intruder) through sniffing or touching and passive social interactions (defined as when the intruder initiated contact with the subject dam) through sniffing or touching. After testing, the female intruder was removed. 11 WT, 8 HET, and 8 HOM control dams and 11 WT, 6 HET, and 9 HOM CUMS dams were assessed using the maternal intruder test.

**DTI image acquisition and data processing**

A subset of offspring from control and CUMS-exposed dams were allowed to age to adulthood (8 weeks old) for the diffusion tensor imaging (DTI) and the remainder of the litters were euthanized.

The brains with the intact skulls were scanned and imaged for DTI in collaboration with the Small Animal Imaging Facility at Texas Children’s Hospital and the Small Animal Magnetic Resonance Imaging (MRI) core at Baylor College of Medicine. All DTI scans were acquired on a 9.4 T Bruker Avance Biospec Spectrometer, 21-cm bore horizontal scanner with a 35 mm volume resonator (Bruker BioSpin, Billerica, MA) with Paravision 5.1 software (Bruker Biospin, Billerica, MA). The 3D DTI scan parameters were as follows: Spin echo, b-value = 0 and 1000s/mm2, 20 diffusion directions with one non-diffusion weighted image, TR = 500 ms, TE = 14.8 ms, FOV = 1.5 × 1.0 × 2.0 cm, matrix = 164 × 96 × 96, NEX = 1, δ = 3 ms, Δ = 7 ms.

Scalar maps (fractional anisotropy (FA) and radial diffusivity (RD), etc.) and color maps were computed. Fiber tracking, an MRI-based neuroimaging technique that is used to visualize the orientation, location, and anisotropy of white matter tracts in the brain was carried out on all brains using three distinct criteria. Brains were then segmented into twenty-nine regions, and the mean FA values for each voxel, a unit of graphic information in a three-dimensional space, were computed in the various brain regions. The FA value is used as a standard metric for highly directed diffusion. The anatomical scan and the FA volume maps were used to align the brains to a template brain scan from Johns Hopkins that served as an accompanying segmentation map, a reference map of brain sub-regions, using DTI Studio (H. Jiang, S. Mori; Johns Hopkins University, www.cmrm.med.jhmi.edu or www.mri.kennedykrieger.org) [[9](#_ENREF_9)].

The brains were up-sampled or digitally processed so that the matrix of the acquired data will match the template dimensions. The brains were aligned using a two-step procedure- a rigid alignment followed by a flexible alignment. The aligned brains were segmented using the template segmentation map. The segmentation was applied to the template space FA volume maps to yield “mean FA” values for the various regions. The segmentation map was transformed into each subject space and applied to the FA volume maps in their original subject orientation and resolution. The segmentation data was collected in a spreadsheet and subjected to statistical analysis to look for significant differences across the groups.

Initially, the segmentation into the areas pre-defined by the existing Hopkins region-of-interest (ROI) map did not reveal areas of substantial significance. To obtain a finer-grained analysis, software was written using MatLab (Mathworks, Natick, MA) to carry out a direct voxel-to-voxel comparison across the four groups using the 2-way ANOVA analysis on the FA maps. This was done in template-space where there is direct alignment among the brains. This revealed significant differences in the region of the stratum radiatum. This region was outlined as a ROI based on a cut-off of *P* < 0.01 and included isolated voxels within the region of higher *P* values, due to noise. This ROI was then transformed into the subject space for each mouse and average FA values computed for the region.

**REFERENCES**

1. Uchida S, Hara K, Kobayashi A, Otsuki K, Yamagata H, Hobara T, et al. Epigenetic status of Gdnf in the ventral striatum determines susceptibility and adaptation to daily stressful events. Neuron. 2011;69(2):359-72. doi: 10.1016/j.neuron.2010.12.023. PubMed PMID: 21262472.

2. Pardon M, Gerardin P, Joubert C, Perez-Diaz F, Cohen-Salmon C. Influence of prepartum chronic ultramild stress on maternal pup care behavior in mice. Biological psychiatry. 2000;47(10):858-63. PubMed PMID: 10807958.

3. Agnish ND, Keller KA. The rationale for culling of rodent litters. Fundamental and applied toxicology : official journal of the Society of Toxicology. 1997;38(1):2-6. PubMed PMID: 9268601.

4. Deacon RM. Assessing nest building in mice. Nat Protoc. 2006;1(3):1117-9. doi: 10.1038/nprot.2006.170. PubMed PMID: 17406392.

5. Jensen Pena C, Champagne FA. Implications of temporal variation in maternal care for the prediction of neurobiological and behavioral outcomes in offspring. Behavioral neuroscience. 2013;127(1):33-46. doi: 10.1037/a0031219. PubMed PMID: 23398440; PubMed Central PMCID: PMC3947603.

6. Chourbaji S, Hoyer C, Richter SH, Brandwein C, Pfeiffer N, Vogt MA, et al. Differences in mouse maternal care behavior - is there a genetic impact of the glucocorticoid receptor? PloS one. 2011;6(4):e19218. doi: 10.1371/journal.pone.0019218. PubMed PMID: 21552522; PubMed Central PMCID: PMC3084270.

7. Macbeth AH, Stepp JE, Lee HJ, Young WS, 3rd, Caldwell HK. Normal maternal behavior, but increased pup mortality, in conditional oxytocin receptor knockout females. Behavioral neuroscience. 2010;124(5):677-85. doi: 10.1037/a0020799. PubMed PMID: 20939667; PubMed Central PMCID: PMC3175421.

8. Martin-Sanchez A, Valera-Marin G, Hernandez-Martinez A, Lanuza E, Martinez-Garcia F, Agustin-Pavon C. Wired for motherhood: induction of maternal care but not maternal aggression in virgin female CD1 mice. Front Behav Neurosci. 2015;9:197. doi: 10.3389/fnbeh.2015.00197. PubMed PMID: 26257621; PubMed Central PMCID: PMC4512027.

9. Jiang H, van Zijl PC, Kim J, Pearlson GD, Mori S. DtiStudio: resource program for diffusion tensor computation and fiber bundle tracking. Computer methods and programs in biomedicine. 2006;81(2):106-16. doi: 10.1016/j.cmpb.2005.08.004. PubMed PMID: 16413083.


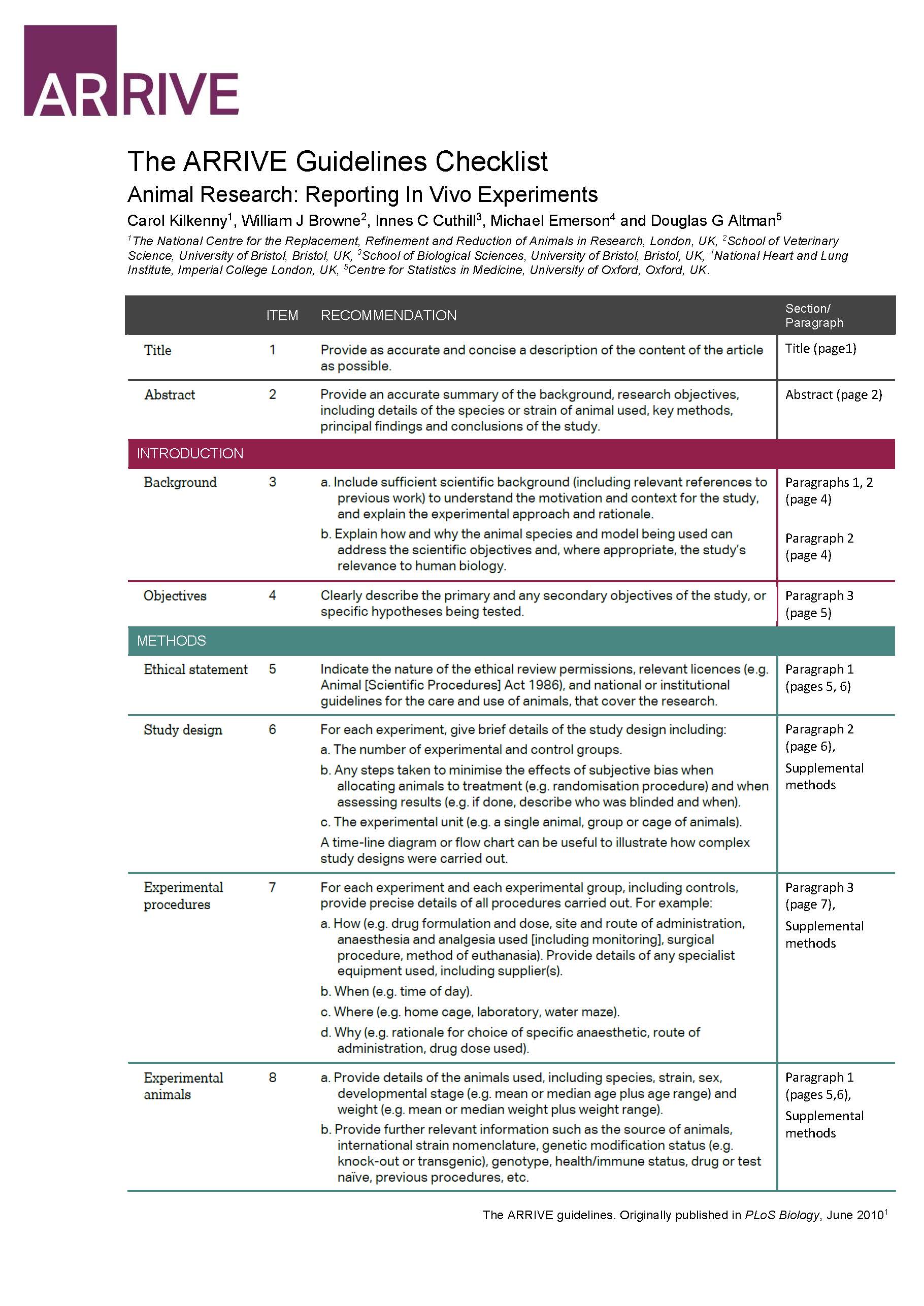


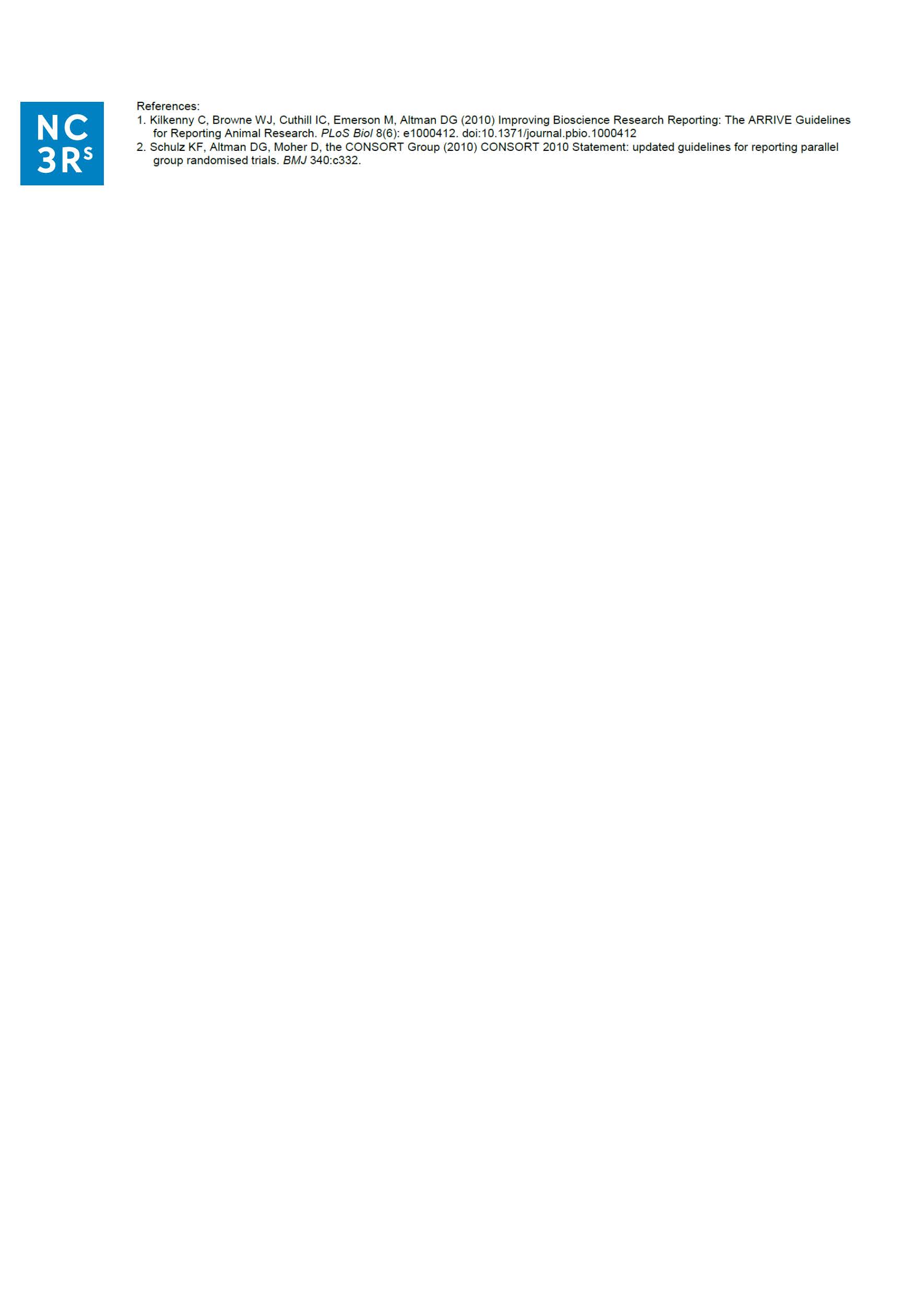

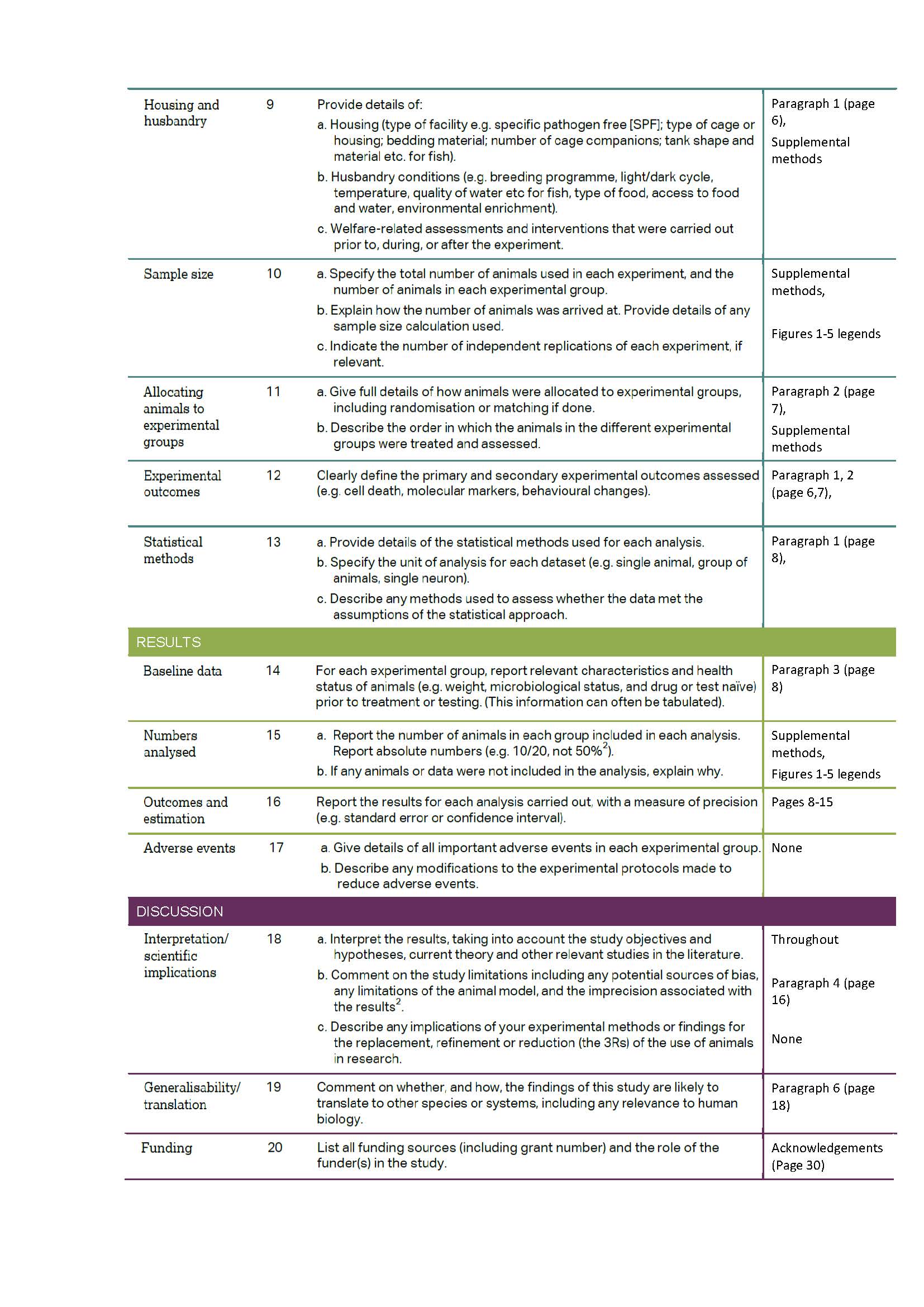

Supplement: S1 File — This file contains all supplemental materials and methods. (DOCX) [file pone.0224876.s003.docx]
