# Supplementary material for: Maternal stress in Shank3ex4-9 mice increases pup-directed care and alters brain white matter in male offspring
Source: PLoS One. 2019 Nov 8;14(11):e0224876. doi: 10.1371/journal.pone.0224876 (PMC6839842; doi:10.1371/journal.pone.0224876)

| **Additional offspring brain regions analyzed by DTI** | **Statistical effects** | | |
| --- | --- | --- | --- |
| Regions analyzed | Genotype | Exposure | G×E interaction |
| Hippocampus | ns | ns | ns |
| Caudate Putamen | ns | ns | ns |
| Lateral Globus Pallidus | ns | ns | ns |
| Ventricles | ns | ns | ns |
| Superior Colliculus | ns | ns | ns |
| Cerebellum | ns | ns | ns |
| Thalamus | ns | ns | ns |
| Hypothalamus | ns | ns | ns |
| Nosebulb | ns | ns | ns |
| Inferior Colliculus | ns | ns | ns |
| Internal Capsule | ns | ns | ns |
| Anterior Commissure | ns | ns | ns |
| Fimbria | ns | ns | ns |
| Corpus Callosum & External Capsule | ns | ns | ns |
| Periaqueductal grey | ns | ns | ns |
| Cingulum | ns | ns | ns |
| Claustrum | ns | ns | ns |
| Dorsal & Ventral Endopiriform nucleus | ns | ns | ns |
| Stria Terminalis | ns | ns | ns |
| Optic Tract | ns | ns | ns |
| Fasciculus retroflexus | ns | ns | ns |
| Stria Medularis | ns | ns | ns |
| Mammillothalamic Tract | ns | ns | ns |
| Fornix | ns | ns | ns |
|  | ns=non-significant for a *P* ≤ 0.05 | | |

**S1 Table: Most offspring brain regions analyzed by DTI do not show significant differences between groups.** Results of diffusion tensor imaging (DTI) conducted in 24 additional brain regions of offspring from HET dams with and without CUMS exposure (Control/CUMS). The number of male offspring in each group was Control (5 WT, 4 HOM) and CUMS (2 WT, 3 HOM).

**References**

1. Uchida S, Hara K, Kobayashi A, Otsuki K, Yamagata H, Hobara T, et al. Epigenetic status of Gdnf in the ventral striatum determines susceptibility and adaptation to daily stressful events. Neuron. 2011;69(2):359-72. doi: 10.1016/j.neuron.2010.12.023. PubMed PMID: 21262472.

2. Pardon M, Gerardin P, Joubert C, Perez-Diaz F, Cohen-Salmon C. Influence of prepartum chronic ultramild stress on maternal pup care behavior in mice. Biological psychiatry. 2000;47(10):858-63. PubMed PMID: 10807958.

3. Agnish ND, Keller KA. The rationale for culling of rodent litters. Fundamental and applied toxicology : official journal of the Society of Toxicology. 1997;38(1):2-6. PubMed PMID: 9268601.

4. Deacon RM. Assessing nest building in mice. Nat Protoc. 2006;1(3):1117-9. doi: 10.1038/nprot.2006.170. PubMed PMID: 17406392.

5. Jensen Pena C, Champagne FA. Implications of temporal variation in maternal care for the prediction of neurobiological and behavioral outcomes in offspring. Behavioral neuroscience. 2013;127(1):33-46. doi: 10.1037/a0031219. PubMed PMID: 23398440; PubMed Central PMCID: PMC3947603.

6. Chourbaji S, Hoyer C, Richter SH, Brandwein C, Pfeiffer N, Vogt MA, et al. Differences in mouse maternal care behavior - is there a genetic impact of the glucocorticoid receptor? PloS one. 2011;6(4):e19218. doi: 10.1371/journal.pone.0019218. PubMed PMID: 21552522; PubMed Central PMCID: PMC3084270.

7. Macbeth AH, Stepp JE, Lee HJ, Young WS, 3rd, Caldwell HK. Normal maternal behavior, but increased pup mortality, in conditional oxytocin receptor knockout females. Behavioral neuroscience. 2010;124(5):677-85. doi: 10.1037/a0020799. PubMed PMID: 20939667; PubMed Central PMCID: PMC3175421.

8. Martin-Sanchez A, Valera-Marin G, Hernandez-Martinez A, Lanuza E, Martinez-Garcia F, Agustin-Pavon C. Wired for motherhood: induction of maternal care but not maternal aggression in virgin female CD1 mice. Front Behav Neurosci. 2015;9:197. doi: 10.3389/fnbeh.2015.00197. PubMed PMID: 26257621; PubMed Central PMCID: PMC4512027.

9. Jiang H, van Zijl PC, Kim J, Pearlson GD, Mori S. DtiStudio: resource program for diffusion tensor computation and fiber bundle tracking. Computer methods and programs in biomedicine. 2006;81(2):106-16. doi: 10.1016/j.cmpb.2005.08.004. PubMed PMID: 16413083.


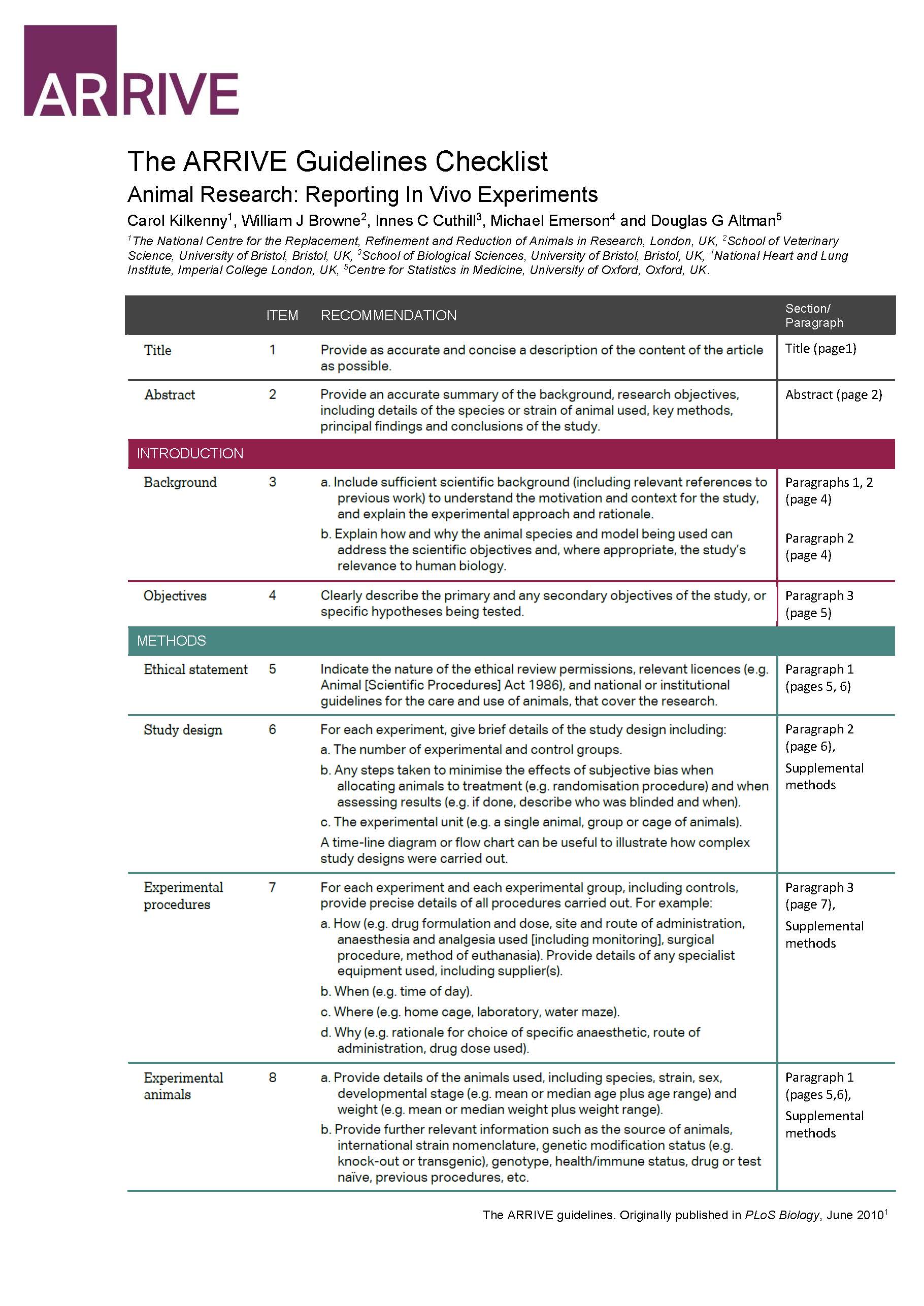


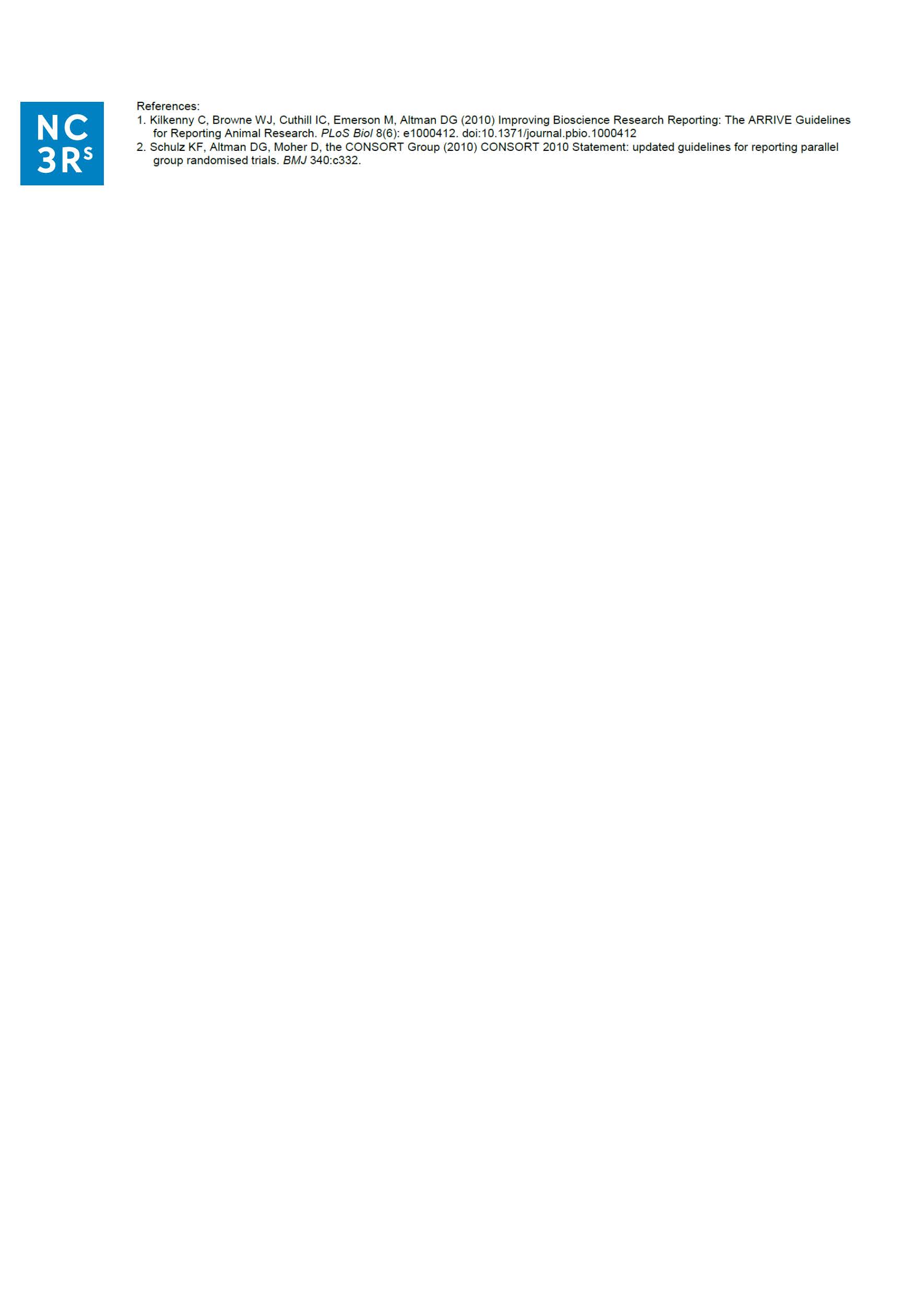

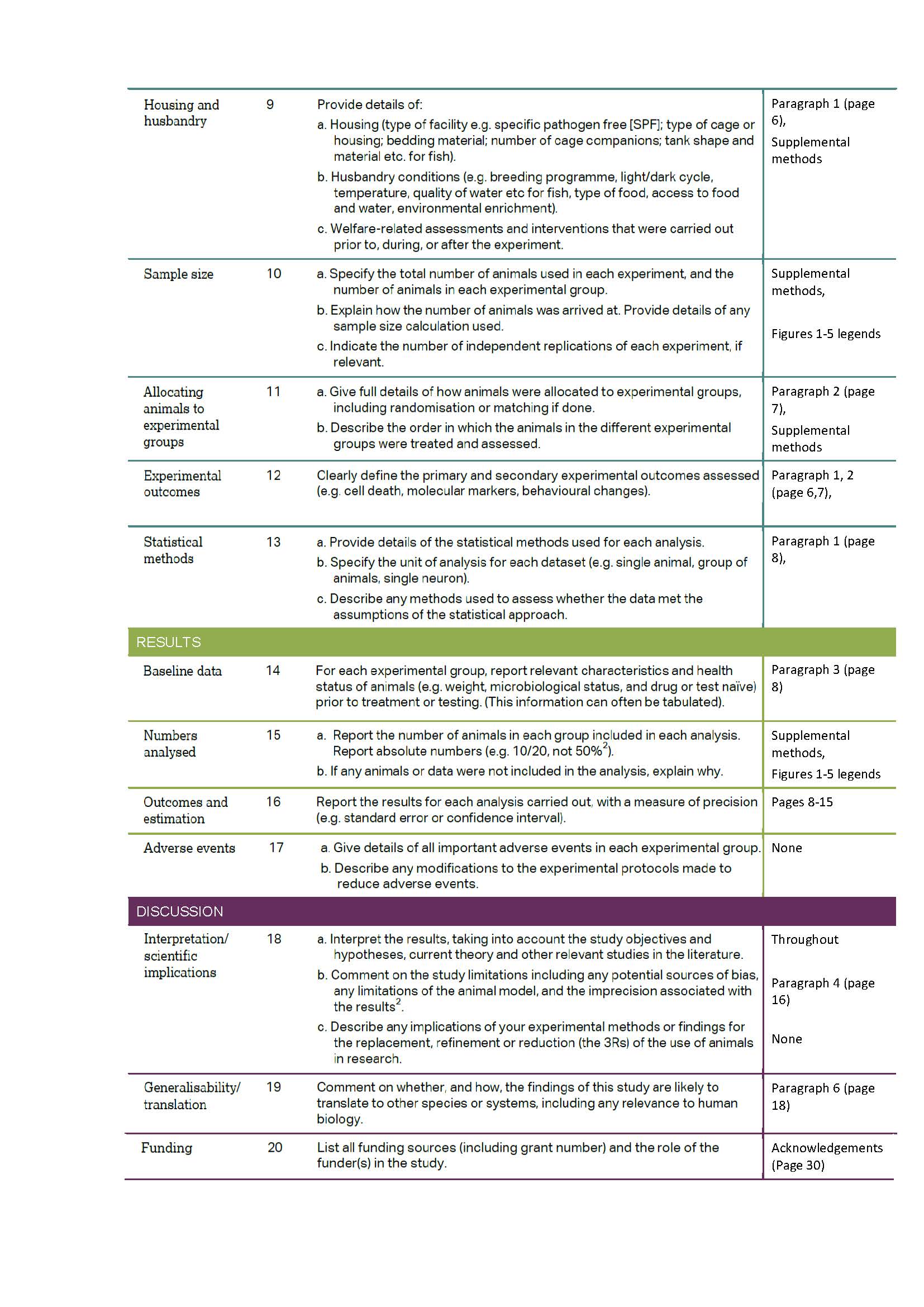

Supplement: S1 Table — Results of diffusion tensor imaging (DTI) conducted in 24 additional brain regions of offspring from HET dams with and without CUMS exposure (Control/CUMS). The number of male offspring in each group was Control (5 WT, 4 HOM) and CUMS (2 WT, 3 HOM). (DOCX) [file pone.0224876.s004.docx]
